# Unveiling Brass-Doped CoSb_3_-Based Thermoelectric Materials Using Solid-State Reaction

**DOI:** 10.3390/ma18173928

**Published:** 2025-08-22

**Authors:** Dan Zhao, Yonghua Ji, Bingke Qin, Jiaxin Fan, Xiaodong Lv, Run Huang

**Affiliations:** 1 School of Chemistry and Materials Engineering, Liupanshui Normal University, Liupanshui 553004, China; zhaodan0910@126.com (D.Z.);; 2 School of Chemical Engineering and Technology, China University of Mining and Technology, Xuzhou 221116, China; 3 College of Materials and Metallurgy, Guizhou University, Guiyang 550025, China

**Keywords:** solid-phase reaction, thermoelectric materials, brass-doped, microstructure

## Abstract

Skutterudite (CoSb_3_)-based thermoelectric materials are regarded as one of the most promising candidates for mid-temperature commercial thermoelectric applications, thanks to their excellent electrical performance and alloy-based attributes. By utilizing techniques such as doping, microstructure design, and high-temperature solid-state reactions, synthesis of Brass_x_/Co_4_Sb_11.5_Te_0.5_ (x = 0.1, 0.3, 0.5, 0.7, representing wt%) in composite form can be rapidly achieved. XRD analysis indicates that the prepared Brass_x_/Co_4_Sb_11.5_Te_0.5_ samples primarily exhibit the CoSb_3_ crystal structure, with the formation of minor impurity phases such as Cu_13_Te_7_ and ZnTe. SEM and EDS analyses reveal that the sample is composed of nanoscale equiaxed grains, some of which are micrometer in size, with a large number of microporous structures distributed uniformly, forming abundant grain boundaries. By co-doping with brass and tellurium (Te), the carrier concentration can be effectively regulated, thereby enhancing the power factor of CoSb_3_-based thermoelectric materials. Meanwhile, the introduction of nanostructures, grain boundaries, and defects optimizes the microstructure of the samples, leading to a reduction in the lattice thermal conductivity of the CoSb_3_-based thermoelectric materials. At a testing temperature of 781 K, Brass_0.1_/Co_4_Sb_11.5_Te_0.5_ achieved a maximum power factor of 1.86 mW·m^−1^·K^−2^, a minimum lattice thermal conductivity of 1.02 W/(mK), and a maximum thermoelectric figure of merit *ZT* of 0.81.

## 1. Introduction

With the increasing severity of energy shortages and environmental issues, the development of green, renewable energy sources and efficient energy conversion technologies has become a worldwide focus of research [1,2]. The thermoelectric conversion technology can achieve the direct conversion between thermal energy and electrical energy. Thermoelectric devices have the advantages of being noise-free, vibration-free, without mechanical components, requiring no medium, simple in structure, and stable in performance [3,4,5,6]. The conversion efficiency of thermoelectric devices is related to the thermoelectric performance of the materials. The dimensionless figure of merit (*ZT*, defined as *ZT* = *S*^2^*σT*/*κ*) is a key indicator for evaluating the thermoelectric properties of materials, where *S*, *σ*, and *κ* represent the Seebeck coefficient, electrical conductivity, and thermal conductivity of the thermoelectric material at a given absolute temperature T, respectively. The power factor (*PF* = *S*^2^*σ*) represents the electrical transport properties of a material. Thermal conductivity *κ* consists of two parts: carrier scattering (electron thermal conductivity *κ_e_*) and lattice phonon vibration scattering (lattice thermal conductivity *κ_L_*), representing the thermal transport properties of a material. According to the Wiedemann–Franz law *(κ_e_* = *LσT*), the lattice thermal conductivity (*κ_L_*) of a material can be regulated relatively independently [7,8]. These parameters are mutually coupled, making it difficult to individually regulate and optimize a specific performance parameter. Therefore, reducing the lattice thermal conductivity while simultaneously optimizing both the electronic and phonon transport properties is an effective strategy for preparing thermoelectric materials with high *ZT* values [9,10,11].

CoSb_3_-based skutterudite is a narrow-bandgap semiconductor with high carrier mobility and excellent electrical transport properties. However, the covalent bonding between Co and Sb, as well as the weak covalent bonds between Sb atoms, result in relatively high lattice thermal conductivity [2,12]. Nevertheless, skutterudites, owing to their unique cage-like structure and their “phonon-glass electron-crystal (PGEC)” characteristics, are considered among the most promising thermoelectric materials for commercial applications in the mid-temperature range [13,14]. The main approaches to optimizing the thermoelectric performance of CoSb_3_-based skutterudite materials include doping or substituting atoms at the Co or Sb sites to introduce point defects, thereby modifying the electrical transport properties and electron–phonon scattering mechanisms of the material [10,15,16]. Filling the lattice voids with suitable atoms can not only regulate the carrier concentration and Fermi level of the material, but also effectively scatter low-frequency phonons through the vibrations of the filler atoms, thereby significantly reducing the lattice thermal conductivity and further optimizing the thermoelectric performance of the material [17,18]. During the preparation process, the material undergoes nanostructuring of its grain structure. The nanostructure can form abundant grain boundaries, which enhance the effect of phonon scattering and reduce the thermal conductivity of the material [19,20,21,22].

Brass is a copper–zinc alloy material with excellent mechanical properties, corrosion resistance, and good hot and cold workability. It typically contains small amounts of elements such as silicon (Si), aluminum (Al), phosphorus (P), and iron (Fe). Brass is widely used in the manufacturing of various precision and complex components that require high strength and wear resistance [23,24,25]. Studies have shown that introducing Cu nanoparticles into a Co_0.91_Ni_0.09_Sb_3_ matrix via a hydrothermal method combined with spark plasma sintering (SPS) can enhance the *ZT* value by approximately 57% compared with pristine Co_0.91_Ni_0.09_Sb_3_ [26]. Copper-doped skutterudites with the composition Cu_x_Co_4_Sb_12_ can be synthesized through a solid-state reaction method; at 578 K, the Cu_0.1_Co_4_Sb_12_ sample exhibits the *ZT* value of 0.24 [27]. (Cu,Te) co-doped CoSb_3_ thermoelectric compounds were synthesized via the high-pressure high-temperature (HPHT) method, and the Cu_0.3_Co_4_Sb_11.5_Te_0.5_ sample exhibited the maximum *ZT* value of 0.77 at 770 K [28]. In addition, the multi-scale synergistic regulation effect of Zn in Ga-doped PbTe systems has been confirmed to effectively increase the electron carrier concentration while significantly suppressing the lattice thermal conductivity [29]. Si, as an abundant element in the Earth’s crust and non-toxic, possesses a high power factor. Most metal silicides (such as Mg_2_Si, MnSi_X_, CrSi_2_, and β-FeSi_2_) exhibit moderate melting points and band gaps, demonstrating excellent potential for thermoelectric applications in the mid-temperature range [30,31]. Studies have shown that when the Al doping concentration is 2 mol%, the electrical conductivity of the pristine ZnO nanostructure is significantly improved while its thermal conductivity is reduced. The aluminum-doped ZnO nanostructure exhibits optimal thermoelectric performance at this doping level [32]. By doping Phosphorus at the Si sites, P-doped Ta_4_SiTe_4_ polycrystalline bulks were synthesized, leading to an increase in carrier concentration, enhanced electrical conductivity, and a significant improvement in the power factor.

The lattice thermal conductivity of Ta_4_Si_1-x_P_x_Te_4_ bulks is below 1.2 W·m^−1^·K^−1^. At 300 K, the thermoelectric figure of merit of polycrystalline Ta_4_Si_0_._995_P_0_._005_Te_4_ reaches 0.18, which is twice that of the pristine Ta_4_SiTe_4_ polycrystalline sample [33]. On the other hand, Cu_2_Te belongs to the “phonon-liquid electron-crystal” (PLEC) class of materials and is prone to excessive carrier concentration due to copper vacancies. Fe doping can control the high carrier concentration, restrict copper ion diffusion, and enhance the Seebeck coefficient. The maximum *ZT* value of the Cu_1.97_Fe_0.03_Te sample at 750 K is approximately 0.16 [34]. However, there have been few reports on the use of brass as a dopant in thermoelectric materials. Therefore, employing brass as a dopant not only introduces copper but also enables the trace incorporation of elements such as zinc, silicon, aluminum, phosphorus, and iron, which holds promise for both cost reduction and performance enhancement of CoSb_3_-based thermoelectric materials. The preparation methods can also alter the microstructure of CoSb_3_-based thermoelectric materials, thereby affecting their thermoelectric transport properties. Currently, synthesis techniques include melt growth, melt annealing (also known as solid-state reaction), mechanical alloying, solvothermal methods, melt spinning, spark plasma sintering (SPS), high-pressure high-temperature (HPHT) techniques, self-propagating high-temperature synthesis (SHS), molecular beam epitaxy (MBE), and others [11,13,35,36]. The solid-state reaction method is a classical approach for preparing thermoelectric materials. In this method, the sample is vacuum-sealed and subjected to a solid-state reaction at high temperatures for a certain period, thereby achieving material synthesis [13].

Brass, with its exceptional corrosion resistance, non-magnetic character, and high electrical conductivity [37], is introduced as a novel alloy dopant, co-doped with Te, to engineer CoSb_3_-based composites. This work uncovers the synergistic regulation of crystal defects and carrier concentration by brass and Te, and elucidates their impact on phonon scattering and thermoelectric transport. The findings are expected to provide new theoretical insights and experimental support for optimizing the thermoelectric performance of CoSb_3_ materials and promoting the practical application of skutterudite thermoelectric materials in fields such as waste heat recovery.

## 2. Experimental

4N high-purity Te powder (200 mesh), Co powder, Sb powder, and brass alloy powder (350 mesh) were used as raw materials. The weighed raw materials were loaded into the ball-mill jar at a ball-to-powder ratio of 6:1 and mechanically ball-milled at 580 rpm under a nitrogen atmosphere for 3 h to ensure thorough mixing. After mixing thoroughly, the powder is cold pressed into cylindrical blocks, which are then sealed and assembled using a metal cylinder sintering mold. The samples are placed in a high-temperature atmosphere sintering furnace for solid-state reaction to prepare the sintered samples. Accurately weigh the raw materials according to the chemical formula Brass_x_/Co_4_Sb_11.5_Te_0.5_ (x = 0.1, 0.3, 0.5, 0.7, where x represents wt%).

The powders were milled using a PMQW2 omnidirectional planetary ball mill from Chishun Technology Co., Ltd., Nanjing, China. The samples were prepared using an HMZ-1700-20 vacuum atmosphere sintering furnace from Haoyue Electric Furnace Co., Ltd., Shanghai, China, with a sintering temperature of 923 K and a synthesis time of 45 min. XRD measurements of the samples were performed using a 6100AS X-ray diffractometer (with a diffraction angle range of 20° to 80°) from Shimadzu Corporation, Kyoto, Japan. The microstructural analysis of the samples was conducted using a Zeiss Gemini 300 scanning electron microscope from Carl Zeiss AG, Oberkochen, Germany. The electrical properties of the samples were tested using a Seebeck-resistivity tester (ZEM-3) from Wuhan Jiayi Tong Co., Ltd., Wuhan, China. The density *ρ* was measured using an MDJ-600S electronic densimeter (Lichen Instrument Technology Co., Ltd., Shanghai, China), and the thermal diffusivity (*λ*) was measured using an LFA475 laser flash apparatus (Netzsch Instruments Co., Selb, Germany), and the crystal specific heat capacity (*Cp*) was calculated using the Dulong–Petit law. The thermal conductivity (*κ*) of the samples was then calculated using the formula: *κ* = *λ·C_p_·ρ*. Subsequently, the electronic thermal conductivity (*κ_e_*) was obtained using the Wiedemann–Franz law, *κ_e_* = *LσT*, and the lattice thermal conductivity (*κ_L_*) was calculated using the formula: *κ_L_* = *κ* − *κ_e_*.

## 3. Results and Discussion

### 3.1. Analysis of Phase Composition and Microstructure

**Figure 1:**
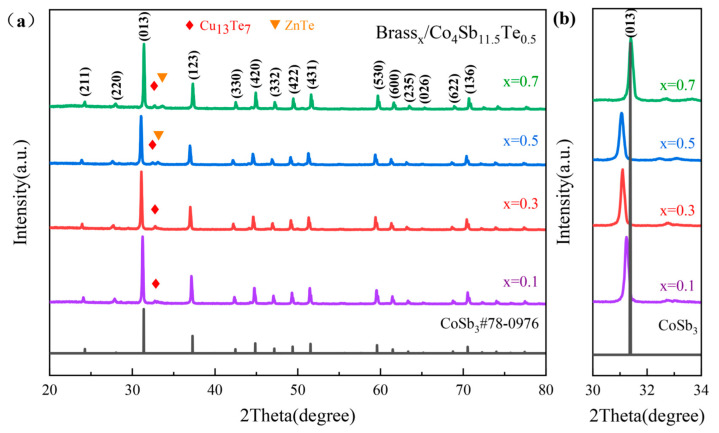
Brass_x_/Co_4_Sb_11.5_Te_0.5_ Samples: (**a**) The XRD spectra, (**b**) the magnified (013) peak.

Figure 1 shows the XRD pattern of the Brass_x_/Co_4_Sb_11.5_Te_0.5_ samples prepared by the solid-state reaction method. XRD analysis indicates that after solid-state reaction sintering, the main characteristic diffraction peaks of all samples with different weight percentages of brass alloy powder match well with those of standard CoSb_3_ (PDF#78-0976). The skutterudite phase is the primary phase in the Brass_x_/Co_4_Sb_11.5_Te_0.5_ materials, with a space group of Im-3, corresponding to a body-centered cubic structure. Compared with the standard CoSb_3_ (PDF#78-0976) reference card, the XRD patterns of the Brass_x_/Co_4_Sb_11.5_Te_0.5_ samples show additional diffraction peaks corresponding to a secondary phase, indicating the presence of minor impurity phases. These peaks were identified as Cu_13_Te_7_ and ZnTe through phase comparison. This phenomenon is mainly attributed to the increased addition of brass alloy powder, which promotes the reaction of Cu, Zn and Te elements during the solid-state reaction process, leading to the formation of the impurity phases Cu_13_Te_7_ and ZnTe. Meanwhile, with the incorporation of brass alloy, the main diffraction peak (013) of the Brass_x_/Co_4_Sb_11.5_Te_0.5_ samples shifts toward lower angles as the doping concentration increases.

**Figure 2:**
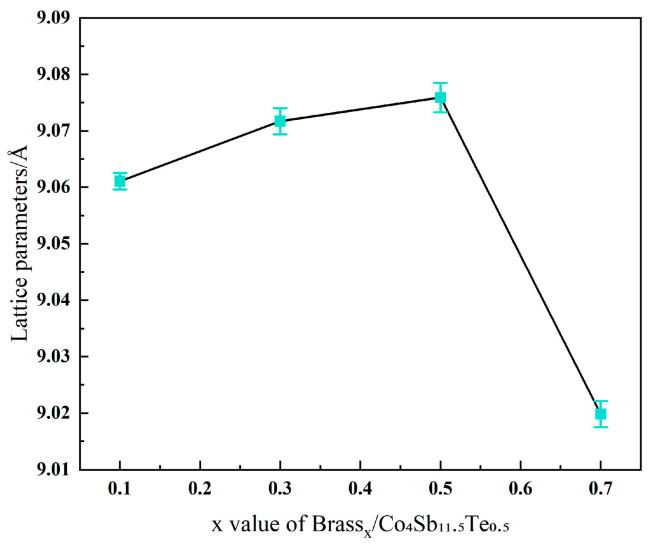
Lattice parameters (including errors) of the Brass_x_/Co_4_Sb_11.5_Te_0.5_ sample.

The shift in the main diffraction peak (013) to lower angles is due to the incorporation of atoms such as Cu, Zn and Te into the skutterudite lattice structure, along with impurity atoms like Cu, Zn and Te entering the lattice voids, which causes lattice expansion. This lattice distortion is beneficial for optimizing the thermal transport properties of the skutterudite phase. However, when x = 0.7, the formation of additional impurity compounds, such as Cu_13_Te_7_ and ZnTe, leads to a reduction in the amount of Te atoms incorporated into the lattice, while the concentration of dopants like Cu and Zn increases. This results in lattice contraction and a decrease in interplanar spacing, causing the main (013) peak to shift toward higher angles. As shown in Figure 2, it can also be confirmed that when x = 0.1–0.5, the lattice parameter of the Brass_x_/Co_4_Sb_11.5_Te_0.5_ sample increases linearly with the mass fraction of brass. When x = 0.7, however, the lattice parameter of Brass_0.7_/Co_4_Sb_11.5_Te_0.5_ decreases again to 9.0198 Å.

**Figure 3:**
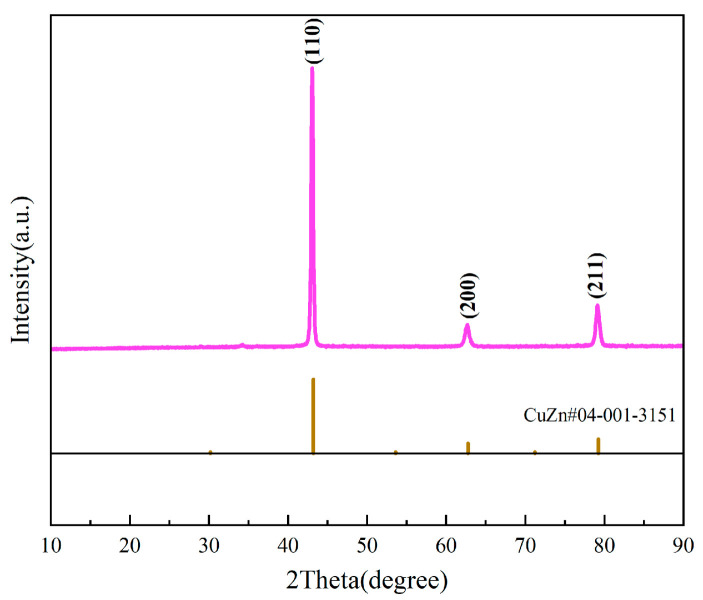
XRD spectrum of Brass Alloy Powder.

No copper elements or copper–zinc compounds were detected in the XRD pattern of the sample. Consequently, we conducted compositional and phase analyses on the raw brass material. The X-ray fluorescence (XRF) scanning test results of the brass alloy powder are shown in Table 1. Due to the manufacturing process and element ratio used by the producer, the results indicate that the Cu and Zn mass ratio in the selected brass alloy powder is close to 1:1. Additionally, trace amounts of impurities such as Si, Fe, Al, and P are also present. The XRF-measured composition has a tolerance of ±0.5%. From the XRF data, the Cu and Zn at.% per formula unit of the introduced sample Brass_x_/Co_4_Sb_11.5_Te_0.5_ were calculated (Table 2). Figure 3 presents the X-ray diffraction (XRD) pattern of the raw brass material. The analysis reveals that the diffraction peaks of the sample are consistent with those of the standard CuZn reference card (PDF#04-001-3151), indicating a crystal structure of Pm-3m. No additional impurity peaks were detected. From the phase analysis, it can be observed that within the doping concentration range, copper elements and the majority of zinc elements have incorporated into the crystal structure of skutterudite, without significant segregation occurring.

**Figure 4:**
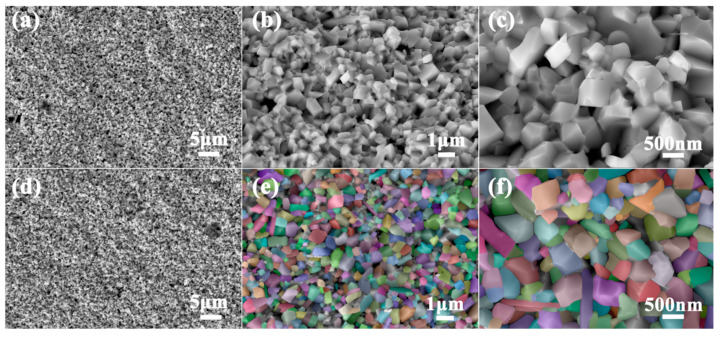
Cross-section SEM images of Brass_x_/Co_4_Sb_11.5_Te_0.5_: (**a**–**c**) x = 0.1; (**d**–**f**) x = 0.3.

**Figure 5:**
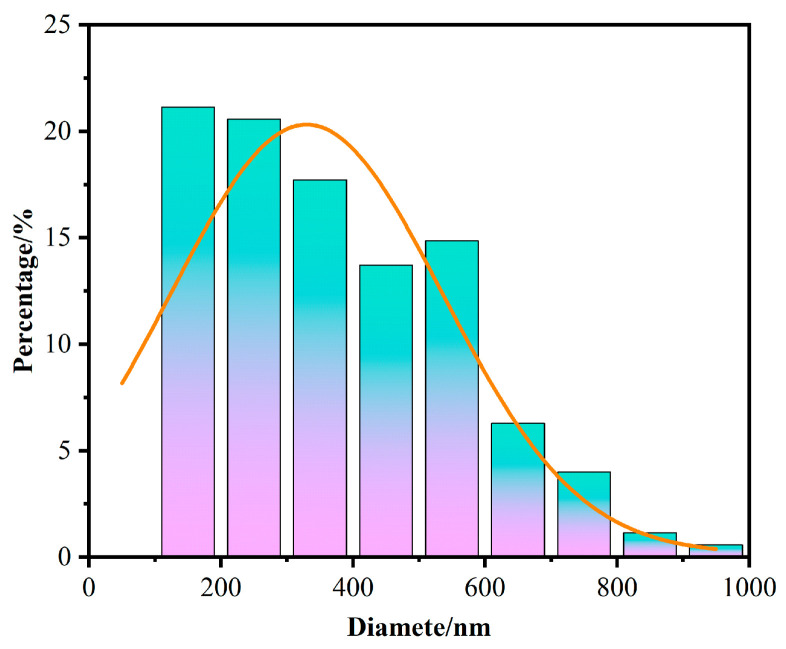
Particle Size Distribution Histogram of Sample Brass_0.3_/Co_4_Sb_11.5_Te_0.5_.

**Figure 6:**
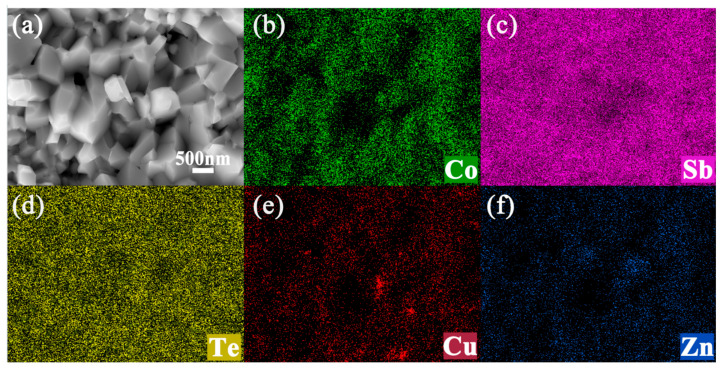
EDS mapping of Brass_0.3_/Co_4_Sb_11.5_Te_0.5_: (**a**) the scanned surface, (**b**–**f**) the distribution of Co, Sb, Te, Cu, and Zn element.

The cross-sectional scanning electron microscope (SEM) images of the samples Brass_0.1_/Co_4_Sb_11.5_Te_0.5_ and Brass_0.3_/Co_4_Sb_11.5_Te_0.5_ are shown in Figure 4. As shown in the figure, the microstructure of the sample consists of equiaxed grains with multiple size scales. The majority of the grains are in the nanometer range, while some have diameters in the micrometer range. Particle size distributions were quantified in ImageJ (version Fiji) and images were pseudo-colored in PS (version 2019). According to Table 3 and Figure 5, the average grain size of the sample Brass_0.3_/Co_4_Sb_11.5_Te_0.5_ is 371 nm. A large number of micropores of varying sizes are uniformly distributed at the grain boundaries, with pore diameters of 1 μm or smaller. The grain refinement and the presence of micro-porous structures contribute to the formation of abundant grain boundaries. Figure 6 shows the EDS analysis map of the Brass_0.3_/Co_4_Sb_11.5_Te_0.5_ grains. EDS analysis reveals that the distribution of elements such as Co, Sb, Zn, and Te is relatively uniform, whereas Cu shows a tendency to accumulate. This may be due to the agglomeration of the impurity phase Cu_13_Te_7_. Microscopic morphology analysis of the Brass and Te composite-doped CoSb_3_ skutterudite-based thermoelectric materials shows that the generated microporous structure, nanometer-sized grains, and grain boundaries, which effectively scatter heat-carrying phonons, shorten their mean free paths, and suppress phonon transport, thereby reducing the thermal conductivity of the samples [7]. In addition, interfacial energy barriers may act as filters for low-energy carriers, leading to an enhanced Seebeck coefficient [38,39].

### 3.2. Analysis of Electrical Transport Properties

**Figure 7:**
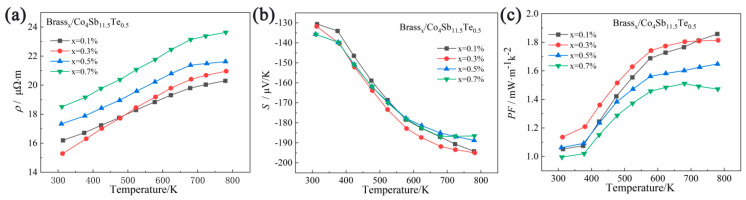
Test temperature dependence of (**a**) resistivity, (**b**) Seebeck coefficient, and (**c**) power factor of Brass_x_/Co_4_Sb_11.5_Te_0.5_.

Figure 7a shows the relationship between the electrical resistivity and temperature for the Brass_x_/Co_4_Sb_11.5_Te_0.5_ samples. As seen in the figure, with the increasing Brass composite doping amount, the electrical resistivity of the samples gradually increases near room temperature. The resistivity of all samples shows an increasing trend with temperature, followed by gradual flattening, exhibiting characteristics of a heavily doped semiconductor. Furthermore, the presence of a large number of pores strongly scatters charge carriers, hindering electron transport and leading to an increase in electrical resistivity. At a test temperature of 312 K, the Brass_0.3_/Co_4_Sb_11.5_Te_0.5_ sample achieves the lowest room-temperature resistivity of 15.28 μΩ·m. At a test temperature of 781 K, the Brass_0.1_/Co_4_Sb_11.5_Te_0.5_ sample achieves the lowest resistivity at this temperature, 20.30 μΩ·m. This value is significantly lower than the lowest resistivity of 165.28 mΩ·cm for undoped skutterudite prepared by the solid-state reaction method [40], and also lower than the lowest resistivity of 36 μΩ·m for undoped CoSb_3_ synthesized by high-temperature high-pressure methods combined with spark plasma sintering [41]. This suggests that by using the technique of co-doping Brass and Te in the composite CoSb_3_, the electrical resistivity of the skutterudite-based thermoelectric materials can be tuned to a lower level through the solid-state reaction method.

The variation in the Seebeck coefficient with temperature for the Brass_X_/Co_4_Sb_11.5_Te_0.5_ sample is shown in Figure 7b. As the testing temperature increases, the absolute value of the Seebeck coefficient for all samples increases with temperature and tends to stabilize after 680 K. When x = 0.1, the sample shows the largest increase in the absolute value of the Seebeck coefficient; when x = 0.7, the absolute value of the Seebeck coefficient decreases regularly after 680 K. This phenomenon is mainly attributed to the increased formation of secondary phases Cu_13_Te_7_ and ZnTe with higher brass doping content, where the scattering effects caused by these secondary phases and other microstructural defects contribute to this behavior. When the testing temperature is 781 K, the sample Brass_0_._1_/Co_4_Sb_11.5_Te_0.5_ exhibits the maximum absolute Seebeck coefficient, with a value of 194.22 μV/K.

The power factor of the sample can be calculated from the above resistivity and Seebeck coefficient data, as shown in Figure 7c. As the testing temperature increases, the power factor of the Brass_x_/Co_4_Sb_11.5_Te_0.5_ sample increases. When the temperature reaches 680 K, the *PF* values of all samples, except for Brass_0_._3_/Co_4_Sb_11.5_Te_0.5_ and Brass_0_._7_/Co_4_Sb_11.5_Te_0.5_, increase with the temperature, while the *PF* values of these two samples decrease as the temperature rises. In the Brass_X_/Co_4_Sb_11.5_Te_0.5_ system, variations in the content of secondary phases such as Cu_13_Te_7_ and ZnTe modify the interfacial density and compositional uniformity, thereby influencing carrier transport pathways. In the temperature range of 300–680 K, thermal excitation enhances carrier concentration, resulting in an increase in the *PF*. However, when the temperature exceeds 680 K, the heterogeneous distribution of secondary phases disrupts the carrier transport network, suppressing further improvement in *PF*. Meanwhile, potential barriers formed at the interfaces between the secondary phases and the Co_4_Sb_11.5_Te_0.5_ matrix exert an energy-filtering effect, effectively blocking low-energy carriers while allowing high-energy carriers to traverse, which enhances the Seebeck coefficient. At elevated temperatures, when the thermal energy approaches or exceeds the barrier height (*E_b_* ≈ *k_B_T*), the efficiency of energy filtering decreases, leading to insufficient Seebeck enhancement. Consequently, the *PF* values of Brass_0_._3_/Co_4_Sb_11.5_Te_0.5_ and Brass_0_._7_/Co_4_Sb_11.5_Te_0.5_ exhibit a decline above 680 K [39,42]. When the testing temperature is 781 K, the sample Brass_0_._1_/Co_4_Sb_11.5_Te_0.5_ achieves the maximum power factor of 1.86 mW·m^−1^K^−2^; this value is approximately 13.4 times higher than that of undoped skutterudite prepared by the solid-state reaction method. The power factor of CoSb_3_ skutterudite-based thermoelectric materials was significantly improved through solid-state reaction and composite doping with Brass and Te.

### 3.3. Analysis of Thermal Transport Properties

**Figure 8:**
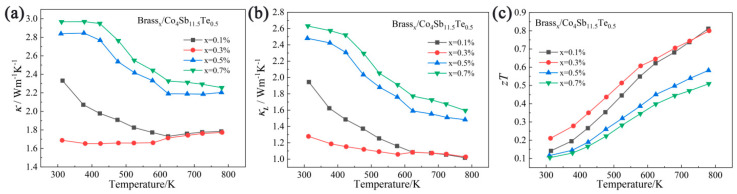
Temperature dependence of thermal transport properties of Brass_X_/Co_4_Sb_11.5_Te_0.5_: (**a**) the total thermal conductivity; (**b**) the lattice thermal conductivity; (**c**) the *ZT* value.

The variation in thermal conductivity with temperature for the Brass_x_/Co_4_Sb_11.5_Te_0.5_ samples synthesized by solid-state reaction is shown in Figure 8a. As shown in the figure, the thermal conductivity of the samples decreases significantly with increasing temperature. When the temperature exceeds 623 K, the decreasing trend slows down for the Brass_0.5_/Co_4_Sb_11.5_Te_0.5_ and Brass_0_._7_/Co_4_Sb_11.5_Te_0.5_ samples, while for the Brass_0_._1_/Co_4_Sb_11.5_Te_0.5_ and Brass_0_._3_/Co_4_Sb_11.5_Te_0.5_ samples, the thermal conductivity shifts from a decreasing to an increasing trend. This slight increase may be attributed to the incorporation of Cu, Zn, and Te into the lattice, which enhances the carrier concentration and consequently increases the electronic contribution to the thermal conductivity.

Figure 8b shows the temperature-dependent lattice thermal conductivity of Brass_x_/Co_4_Sb_11.5_Te_0.5_ samples with different doping concentrations, revealing the complex relationship between phonon transport properties and doping-induced modulation mechanisms within the material. As the temperature increases, the lattice thermal conductivity of all samples shows a decreasing trend, which is consistent with the typical characteristics of lattice thermal conductivity dominated by Umklapp scattering. Umklapp scattering causes the momentum of thermally excited phonons to lose conservation, thereby hindering the transport of thermal energy, which is manifested as a significant decrease in lattice thermal conductivity (*κ_L_*) at high temperatures. However, different mass fractions of brass co-doping exert a significant influence on the lattice thermal conductivity of the samples, thereby leading to corresponding changes in the overall thermal conductivity. When x = 0.1–0.3, the Brass_x_/Co_4_Sb_11.5_Te_0.5_ samples, via the co-doping strategy of brass and Te, introduce multiple crystal structure defects such as Cu_13_Te_7_ secondary phase interfaces, grain refinement, porosity, and lattice distortion, thereby enhancing phonon scattering. With increasing brass doping mass fraction, *κ_L_* decreases further; among them, the Brass_0.3_/Co_4_Sb_11.5_Te_0.5_ sample exhibits the lowest overall lattice thermal conductivity. When x = 0.5–0.7, high-level brass doping is likely to promote the formation of additional impurity phases such as Cu_13_Te_7_ and ZnTe, resulting in the aggregation of secondary phases and a reduction in interface and defect densities. This microstructural ordering decreases the number of phonon scattering centers and extends the phonon mean free path, thereby leading to an increase in lattice thermal conductivity. Consequently, the Brass_0.7_/Co_4_Sb_11.5_Te_0.5_ sample presents the highest overall lattice thermal conductivity.

The key to thermoelectric materials lies in achieving low thermal conductivity and high electrical conductivity in order to enhance the thermoelectric figure of merit (*ZT*) of the material. The thermal conductivity (*κ*) of a material consists of two components: the electronic thermal conductivity (*κ_e_*) and the lattice thermal conductivity (*κ_L_*). The electronic thermal conductivity *κ_e_* arises from heat transport by charge carriers (electrons or holes) and is typically closely related to the material’s electrical conductivity, making it difficult to minimize. Therefore, reducing the lattice thermal conductivity is the primary approach to lowering the overall thermal conductivity. Based on the variation trend of lattice thermal conductivity, the samples exhibit significantly reduced lattice thermal conductivity at high temperatures. At 781 K, the Brass_0_._1_/Co_4_Sb_11.5_Te_0.5_ sample achieves the lowest lattice thermal conductivity of 1.02 W/(m·K). Additionally, at 623 K, the same sample shows the minimum total thermal conductivity of 1.73 W/(m·K).

### 3.4. Analysis of Thermoelectric Figure of Merit (ZT Value)

Figure 8c shows the variation in the *ZT* value with temperature for the Brass_x_/Co_4_Sb_11.5_Te_0.5_ samples. The *ZT* values were calculated using the formula *ZT = S^2^σT/κ* based on the Seebeck coefficient, resistivity, and thermal conductivity data obtained from the above tests. As the testing temperature increases, the *ZT* value of the sample shows a noticeable increasing trend. The *ZT* value of the Brass_0_._1_/Co_4_Sb_11.5_Te_0.5_ sample reaches its maximum at 781 K, with a peak *ZT* of 0.81. Compared with the undoped pure skutterudite prepared by the solid-state reaction method [40], this value of *ZT* has increased by 15.28 times, and by 3.38 times compared with the maximum *ZT* value of single Cu-filled skutterudite [27].

The enhancement in the *ZT* value of the Brass_x_/Co_4_Sb_11.5_Te_0.5_ samples is primarily attributed to the multi-element filling of the brass alloy and the substitution of Te, which together provide additional electrons and significantly reduce the electrical resistivity of the material. Moreover, the optimization of the microstructure, including the incorporation of impurity atoms into the CoSb_3_ lattice and the formation of a ZnTe second phase, led to the scattering of some phonons, thereby reducing the lattice thermal conductivity. This significantly enhanced both the electrical and thermal properties of the CoSb_3_-based skutterudite thermoelectric materials. As a result, the thermoelectric properties of the skutterudite samples were optimized, with the Brass_0_._1_/Co_4_Sb_11.5_Te_0.5_ sample achieving a maximum *ZT* value of 0.81.

## 4. Conclusions

Using the solid-state reaction method, the thermoelectric compound Brass_x_/Co_4_Sb_11.5_Te_0.5_ (x = 0.1, 0.3, 0.5, 0.7, representing wt%) was successfully and rapidly synthesized under sintering conditions of 923 K and a reaction time of 45 min, incorporating a composite secondary phase. The microstructure of the samples consisted of equiaxed grains with multiple sizes, with an average grain size in the nanometer range. A large number of micropores are uniformly distributed at the grain boundaries, and the ZnTe second phase was generated, forming abundant grain boundaries. Co-doping with brass alloy and Te can effectively control the carrier concentration, thereby enhancing the power factor of CoSb_3_-based thermoelectric materials. The introduction of nanostructures, grain boundaries, and defects led to the optimization of the sample’s microstructure, which simultaneously resulted in a lower lattice thermal conductivity. Therefore, by systematically regulating the carrier concentration and lattice thermal conductivity—combined with doping strategies, microstructural design, and solid-state synthesis techniques—the thermoelectric figure of merit (*ZT*) of CoSb_3_-based thermoelectric materials was effectively enhanced. Among them, the Brass_0_._1_/Co_4_Sb_11.5_Te_0.5_ sample exhibited the best thermoelectric transport performance, achieving a maximum power factor of 1.86 mW·m^−1^K^−2^ at 781 K and a maximum thermoelectric figure of merit (*ZT*) of 0.81.

## Figures and Tables

**Table 1:** XRF Scanning Test Results of Brass Alloy Powder.

Component	Test Results (Mass%)	Elemental Spectrum	Intensity
Zn	50.5	Zn-KA	40.1096
Cu	48.8	Cu-KA	38.7163
Si	0.649	Si-KA	0.5156
Al	0.0222	Al-KA	0.0104
P	0.0205	P-KA	0.0163
Fe	0.0201	Fe-KA	0.0160

**Table 2:** Cu and Zn at.% per formula unit of the introduced sample Brass_x_/Co_4_Sb_11.5_Te_0.5_.

Samples	Principal Chemical Elements	Atoms per Formula Unit	Corresponding at.%
Brass_0.1_/Co_4_Sb_11.5_Te_0.5_	Cu	0.01307	0.08166
	Zn	0.01314	0.08214
Brass_0.3_/Co_4_Sb_11.5_Te_0.5_	Cu	0.03920	0.2450
	Zn	0.03942	0.2464
Brass_0.5_/Co_4_Sb_11.5_Te_0.5_	Cu	0.06534	0.4084
	Zn	0.06571	0.4107
Brass_0.5_/Co_4_Sb_11.5_Te_0.5_	Cu	0.09148	0.5717
	Zn	0.09200	0.5750

**Table 3:** Particle Size Statistics of Sample Brass_0.3_/Co_4_Sb_11.5_Te_0.5_.

**Total particles**	175
**Particle size information**	
**Average particle size**	371.133 nm
**Particle size standard deviation**	181.213 nm
**P10**	182.926 nm
**P50**	505.964 nm
**P90**	829.002 nm

## Data Availability

The original contributions presented in this study are included in the article. Further inquiries can be directed to the corresponding author.
